# Documentation of the Development of Various Visuomotor Responses in Typically Reared Kittens and Those Reared With Early Selected Visual Exposure by Use of a New Procedure

**DOI:** 10.3389/fnins.2021.781516

**Published:** 2021-12-10

**Authors:** Katelyn MacNeill, Amber Myatt, Kevin R. Duffy, Donald E. Mitchell

**Affiliations:** Department of Psychology and Neuroscience, Dalhousie University, Halifax, NS, Canada

**Keywords:** visual deprivation, gaze, following, striking, visuomotor, visual acuity, vision

## Abstract

A new procedure was used to study the development of gaze (responses to moving targets or laser spots in normal kittens, those that had been reared in total darkness to 6 weeks of age, and others that received a period of monocular deprivation (MD). Gaze responses were observed to all stimuli in normal kittens at between 25–30 days of age and striking responses occurred on the same day or the next. Despite slow acquisition of spatial vision in the dark reared kittens over 3 months, they were able to follow and even strike at moving visual stimuli within a day of their initial exposure to light. By contrast, for a week following a period of MD, kittens showed no gaze or striking responses to moving stimuli when using their previously deprived eye. The very different profiles of acquisition of visuomotor skills and spatial vision in visually deprived kittens point to a dissociation between the neuronal populations that support these functions.

## Introduction

Discovery of the widespread anatomical and physiological changes observed in the central visual pathways of kittens and monkeys that had received abnormal visual exposure in early postnatal life led naturally to many studies of the consequences for visual function. For the most part, the focus of these studies was placed on the consequences of early selected visual deprivation for visual perceptual abilities linked primarily to neural functioning of the geniculostriate visual pathway. Possible consequences of early visual deprivation for visuomotor behavior received far less attention. Early studies of the effects of monocular and binocular visual deprivation in kittens (Wiesel and Hubel, 1963, 1965) remarked upon the severe consequences for general visuomotor behavior but two later studies (Van Hof-Van Duin, 1976; Vital-Durand et al., 1976) reported that cats reared for extended periods from birth in total darkness to at least 4 months of age were able to move their gaze (combined head and eye movement) to follow moving objects within a day or so. This result was particularly surprising because the dark-reared cats appeared otherwise totally blind in terms of their general behavior. By contrast, assessment of spatial vision such as captured by measurement of visual acuity revealed a very slow recovery that is measured in months and is proportional to the length of prior visual deprivation (Timney et al., 1978; Mitchell, 1988).

In this study we deployed a new method to test longitudinally the development of various visuomotor abilities in normal kittens as well as the recovery of such abilities in kittens dark reared from birth to 6 weeks of age. Preliminary observations were also made on kittens following a period of early monocular deprivation. As with techniques devised to monitor fast changes in spatial vision in normal or selectively visually deprived kittens during development (Mitchell et al., 1976, 1977), methods to monitor longitudinal changes in visuomotor abilities must require little training to be effective. Many existing methods to monitor visually guided behavior frequently require months of training (e.g., Fabre-Thorpe et al., 1984). The method employed here was informed by observations and methodologies that are in widespread use for behavioral studies of rodents (Walsh and Cummings, 1976). In addition to providing new information of the development of following and striking responses to moving solid objects and laser spots in normal kittens, we confirmed and extended previous reports of the fast emergence of gaze responses in dark- reared kittens following their initial exposure to light at a time when tests of visual acuity on the jumping stand indicated that they had no spatial vision. By contrast, following a much shorter period of monocular deprivation, no gaze responses were observed for at least a week following the period of deprivation when the kittens used their previously deprived eye.

## Materials and Methods

### Subjects

Four purpose-bred litters of kittens (*Felis domestica*) from a cat-breeding colony at Dalhousie University served as subjects for this study. The rearing history of all 10 animals is summarized in Table 1. The Dalhousie Committee on Laboratory Animals, in accordance with regulations of the Canadian Council on Animal Care, approved the breeding, rearing and behavioral testing protocols employed for this study. Five kittens from the first litter were assigned to two rearing conditions, a group of 3 kittens that were dark-reared to 6 weeks of age (C114, C115, and C116) and two others that received daily periods of exposure to light (C117 and C118). The latter kittens wore collars around their neck so that from P10 they could be identified in the darkroom and removed with their mother into an illuminated room for 1 h each day over a week. At P17 the daily period of light exposure for these kittens was increased gradually to 2 h and at P33 was increased to 3 h. At 6 weeks, all littermates, including the 3 kittens that hitherto had been dark-reared, were moved permanently with their mother to a regular colony room that was illuminated on a 12:12 h light/dark cycle. The second litter yielded only a single kitten (C119) that was reared with its mother under normal colony lighting conditions from the day of birth. A third litter of 3 kittens born in 2010 was dark-reared from birth to 6 weeks of age and provided data on the recovery of visual acuity with subsequent exposure to light. The remaining data for this paper was obtained from two kittens (C479 and C480) from a litter of 5 born in 2020. These two kittens were reared normally until P30 at which age they received a 3 week period of monocular deprivation by eyelid suture that was followed by either a 7 day period of binocular exposure (C479) or a 10 day period of reverse occlusion (C480). Both kittens were then employed in a separate electrophysiological study.

**TABLE 1:** Information concerning the litter composition, gender and rearing history of the 11 animals of the study.

Animal ID.	Gender	Visual experience
**Litter 1**		
C114	F	DR: P0-P42; LR P42-
C115	F	DR: P0-P42; LR P42-
C116	M	DR: P0-P42; LR P42-
C117	M	DR to P10 then LR 1 h/day P11-17, LR 2 h/day P17-33, LR 3 h/day P33-42
C118.	M	DR to P10 then LR 1 h/day P11-17, LR 2 h/day P17-33, LR 3 h/day P33-42
**Litter 2**		
C119	M	LR from birth
**Litter 3**		
C127	M	DR: P0-42
C128	M	DR: P0-P42
C129	M	DR: P0-P42
**Litter 4**		
C479	F	MD: P30-51; BV: P51-59
C480	F	MD: P31-52; RO P52-62

*M, male; F, female; DR, Dark-reared; LR, light-reared; MD, monocular deprivation; BV, binocular visual input; RO, reverse occlusion; P, postnatal day.*

### Darkroom Facility and Colony Rooms

After being seen by a university veterinarian the first litter was placed within an hour of birth into a darkroom facility that has been described in detail elsewhere (Mitchell, 2013). The facility includes two large interconnected darkrooms, one of which contains a large rearing cage in which kittens and their mother were housed while the other darkroom allowed for cleaning of the first room and for the interchange of clean cages on a regular basis. Both darkrooms were accessed through a series of smaller dark anterooms and hallways separated by a series of entryways and doors. All walls and doors within the darkrooms, surrounding corridors and anterooms were painted black. A large rearing cage (93 cm high, 66.5 cm deep, and 153 cm wide) was used to contain the mother and her kittens. A litter box, dry food and water were available at all times, and cardboard boxes and towels were present for bedding. To entrain a daily circadian rhythm a radio was timed to turn on at 7 am and to turn off at 5 pm, an interval that coincided with the approximate work day of departmental animal care technicians. At 6 weeks of age, the mother and all kittens were relocated to a colony room illuminated with fluorescent lights on a 12:12-h light/dark cycle.

### Behavioral Testing Arena

**FIGURE 1:**
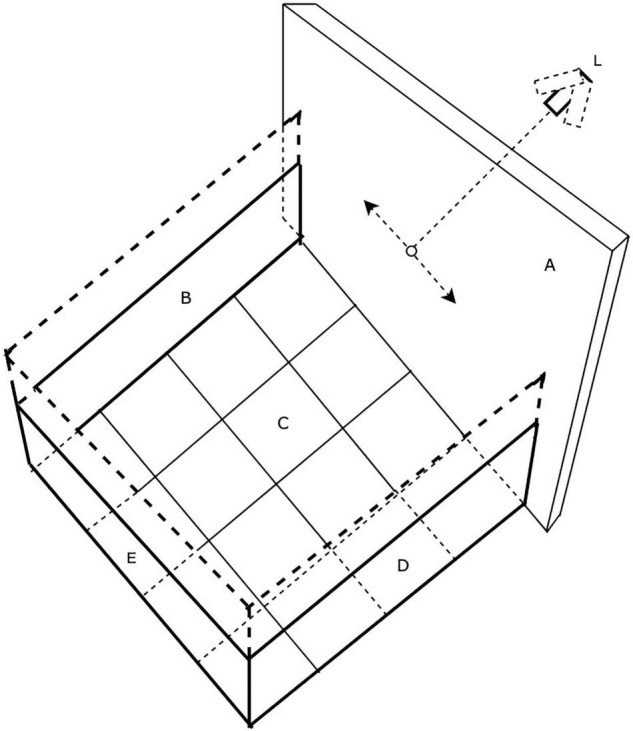
The testing arena. Walls B and C are opaque while wall A is translucent plastic upon which a laser spot could be projected from a laser (L) mounted on a stepping motor and made to move under computer control (L).

**FIGURE 2:**
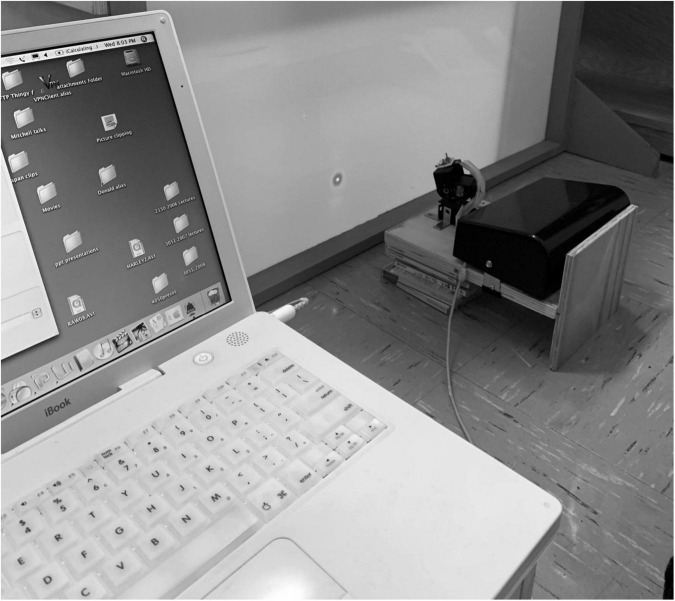
A photograph of the set-up used to project moving laser spots on the wall or floor of the arena. Note the expanded diameter (5 mm) of the laser spot due to scatter by the translucent screen upon which it is projected.

The arena, drawn in schematic fashion in Figure 1, was modeled on the design of those used widely for studies of locomotion in rodents and consisted of a robust plywood square enclosure (80 cm × 80 cm) with an open top. Three of the walls (B) were constructed of 1.6 cm thick plywood while the fourth wall (A) consisted of a translucent white plastic screen onto which laser light spots could be back-projected. For the youngest animals, the wooden walls were only 41 cm high, but as the animals grew and became more mobile, a second set of walls (shown by dashed lines) of the same dimensions were placed on top of the first to double the height to 82 cm. On the cardboard floor (C) of the enclosure a 4 × 4 grid of squares (20 cm × 20 cm) was drawn to allow quantification of the mobility of the animals. The overall mobility of the animals and their gaze responses to moving stimuli were captured by either a tripod mounted video camera (JVC Everio GZ-MG135) camcorder or more recently by a GoPro camera (G) clamped to one of the wooden walls. It was observed that some kittens followed or struck at a moving laser spot with greater frequency if it was projected onto the floor rather than onto the walls of the arena. Consequently, for these animals the arena was modified by replacing the opaque floor with translucent plastic and raising it above the floor so that the laser spot could be projected from below. In addition to use of a hand-held laser, a small laser and assorted electronic components that included a stepping motor were purchased from a toy store and were configured to be controlled by a Mac iBook in order to project a laser spot onto the translucent floor or wall of the arena. The laser spot could be made to move in either linear or sinusoidal fashion at various speeds along the translucent floor or wall of the arena in response to sawtooth or sinusoidal input signals to the stepping motor that controlled the laser. To introduce some unpredictability, the laser spot could be made to make sudden vertical steps during its horizontal motion. The photograph in Figure 2 shows the computer-controlled laser and the laser spot projected onto the lower part of the translucent wall of the arena. Because of diffusion by the translucent plastic wall of the arena, the laser spot had an overall diameter of 5 mm with a central bright region that was 2 mm wide.

### Behavioral Testing in the Arena

As pioneered with rodents, general locomotive behavior was assessed by counts of the number of gridlines on the floor crossed in a fixed amount of time. These measurements were initiated when kittens were 10–12 days old, at a time when their eyelids had been open for a week or less. With the exception of C479 and C480, all animals were video recorded in the arena for a period of 5 min in order to assess their spontaneous behavior. The video records were viewed later to permit numerical scoring of the number of times the kittens crossed the gridlines on the floor of the enclosure with any paw, the number of episodes of sleeping, sitting, play, grooming, crawling or escape attempts. From 25 to 30 days of age, kittens were placed in the arena for videotaped periods of exposure to moving stimuli that included two light spherical pendulums held on strings (one large 55 mm diameter hollow plastic ball that weighed 7 grams; and one small 28 mm diameter solid wooden ball that weighed 9 grams). In addition, responses were assessed to movement of light spots from either a hand-held laser or else the computer-controlled laser described that were shone on either the walls or floor of the arena. In order to assess the responses to more regular movement of a laser spot, a Mac iBook computer was programmed to control the movement of a small laser attached to a stepping motor (see Figure 2). The laser spot was projected onto the translucent screen (A, Figure 1) and moved horizontally at controlled speeds according to either a sinusoidal or sawtooth waveform. In order to introduce a degree of unpredictability to the horizontal path of the spot, it could be programmed to suddenly jump in a vertical direction by the superposition of a vertical sawtooth to the horizontal motion.

In response to moving stimuli, normal kittens at a certain age begin to follow the target by changes of gaze which refer to the coordinated head and eye movements that collectively alter the position of the visual axis (Guitton et al., 1984). Cats have a limited oculomotor range of only ± 25 deg, (Stryker and Blakemore, 1972; Guitton et al., 1980) so that gaze movements are often dominated by motion of the head. Analysis of the changes of gaze from analysis of the video records from the video or GoPro cameras that viewed the kittens from above allowed only for monitoring of changes in head position as a proxy for changes of gaze., Plots of the gaze following responses to the various moving stimuli were plotted from a frame by frame analysis of the video records by use of ImageJ software. Separate plots were made of the angular change of position of the stimulus object (pendulum or laser spot) and of the gaze responses with respect to a fixed reference point on the arena. For gaze responses rotation of the head was measured with respect to an imaginary line orthogonal to the line joining the two ears at its midpoint. The plots of gaze following responses were made independently a decade apart by two of the authors (AM and KM). The plots shown in Figures 6, 7 were made by AM but were very similar to those made earlier by KM for different episodes of following responses for the same animal and day of light exposure.

**FIGURE 3:**
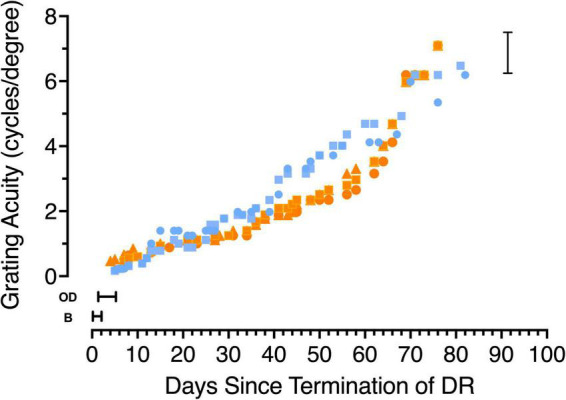
The development of grating acuity in 5 kittens that were reared in total darkness (DR) from birth until 6 weeks of age. Data from the two kittens tested from the first litter are depicted with blue symbols (C115 circles, C116 squares) while orange symbols show data from the second litter (C127 squares, C128 circles and C129 triangles). Periods during which the kittens appeared blind on the jumping stand are indicated by the letter B, while the time during which they were capable of only the ability to discriminate an open from a closed door is indicated by then letters OD. The vertical bracket on the right indicates the range of acuities measured in normal kittens at an equivalent age.

**FIGURE 4:**
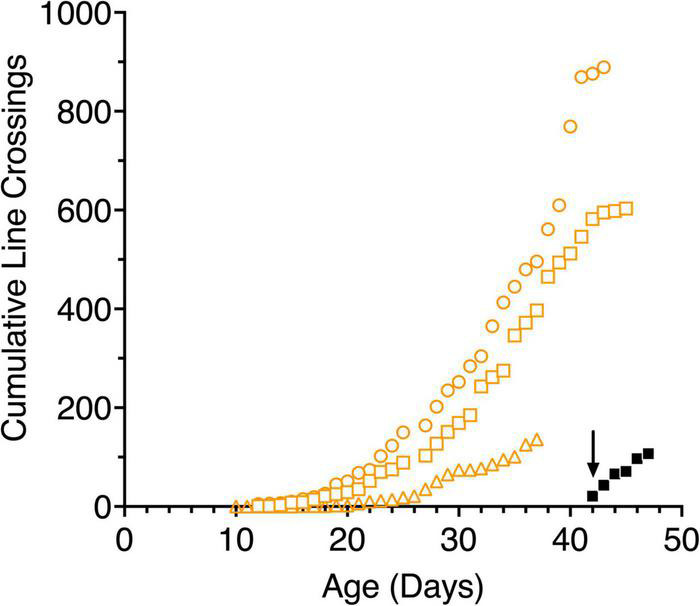
Cumulative line crossings obtained from 5-min trials of 3 light-exposed (open orange symbols: C117 squares, C118 circles, C119 triangles) and a representative dark-reared kitten (C116, filled black symbols) as a function of age. The arrow indicates when the dark reared kitten was first exposed to an illuminated environment.

**FIGURE 5:**
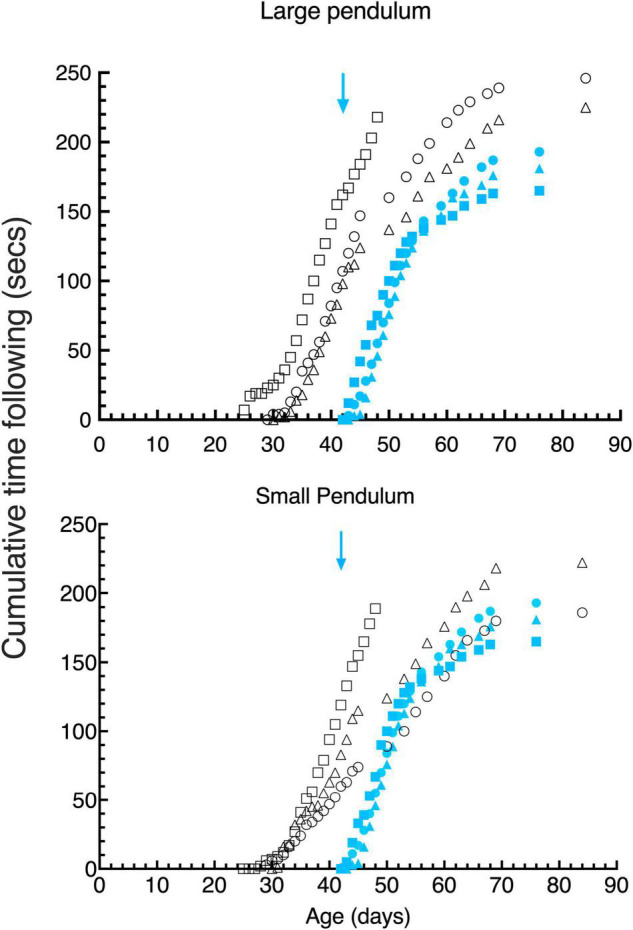
Cumulative gaze- following behavior in daily 15 s episodes as a function of age for large (top) and small (bottom) pendulum swinging pendulums. Data are displayed for 3 light- exposed (open black symbols: C117 triangles, C118 circles, C119 squares) and 3 dark-reared (blue filled symbols: C114 triangles, C115 circles, C116 squares) kittens. The blue arrows indicate the age of the dark-reared kittens when they were first exposed to light.

**FIGURE 6:**
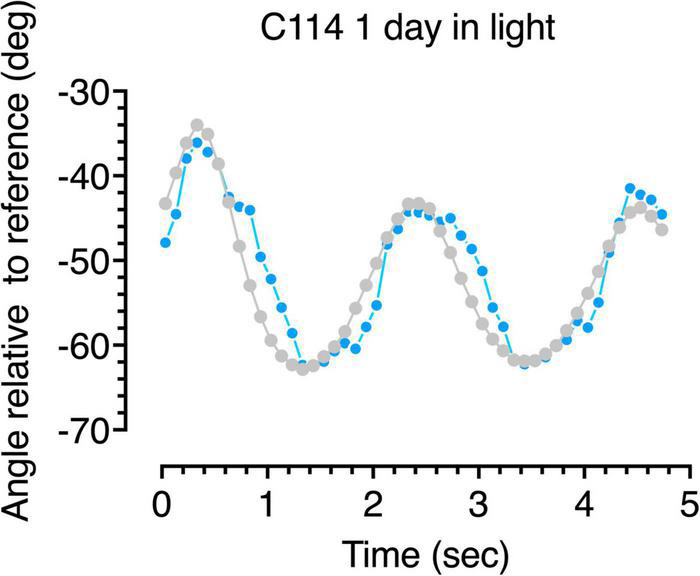
Gaze following responses (blue symbols) to a swinging large pendulum (gray symbols) for a 6-week dark-reared kitten (C114) made on the second day of exposure to light.

**FIGURE 7:**
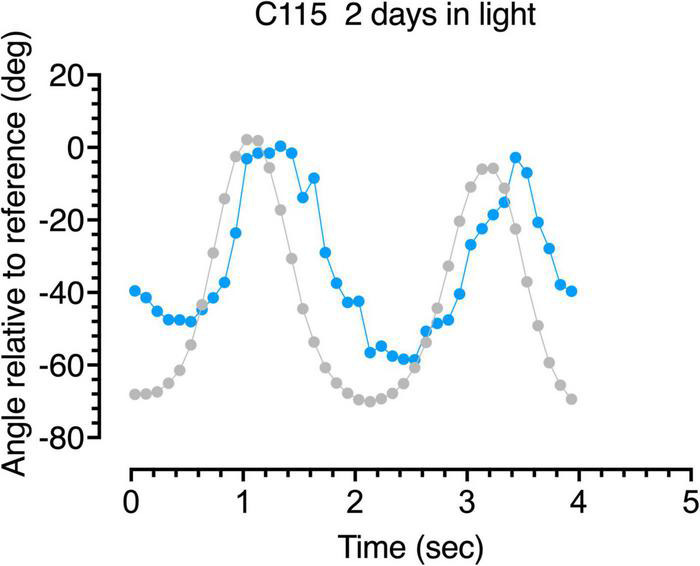
Gaze following responses (blue symbols) to a swinging large pendulum (gray symbols) for a 6-week dark-reared kitten (C115) made on the third day of exposure to light.

### The Jumping Stand

Emergence of spatial vision was documented in 5 of the 6 dark-reared kittens by use of a jumping stand and techniques developed and refined in this laboratory over the last 4 decades (Mitchell et al., 1976, 1977; Mitchell, 2013). Upon removal from the darkroom, kittens exhibited few signs of vision apart from those noted when they were tested in the arena. On the jumping stand they appeared blind as they were unable to discriminate an open door (platform) from an adjacent closed door (on which a large vertical square-wave grating with a period of 32 mm was placed) except by use of tactile cues such as use of their whiskers or paws. From a platform located only 1–2 cm above the divider that separated the open from the closed door they reached out very tentatively to either side in an attempt to locate a surface (the closed door) onto which they could step. Once the animals were able to jump onto the closed door from a few centimeters above it, an ability that we define operationally as an open door discrimination (OD) that may reflect only the ability to make discriminations on the basis of luminance, the kittens were tested formally for the ability to discriminate form information in the manner outlined in many previous papers but summarized by Mitchell (2013). This ability was assessed by closing the open door and placing a horizontal grating with the same period as the vertical grating on the adjacent door. Once the animal could jump correctly to the positive (vertical) grating, it was possible to test acuity by a combination of progressive increases in the spatial frequency of the gratings, and by raising the jumping height until discrimination was not possible. Because the animals had been housed in the darkroom since birth, there was no opportunity to familiarize or train the animals on the jumping stand before they were removed to an illuminated colony room at 6 weeks of age. Notwithstanding this delay in training and the slow emergence of visual guided behavior on the jumping stand, animals learned the essential discrimination task between a vertical and a horizontal grating within a day or two following the ability to detect a closed door (OD discrimination). All tests of vision were made binocularly.

## Results

### Dark-Reared Animals

To set the stage for a description of the results from tests of mobility, visual following, and striking at moving objects, it is important to highlight the slow emergence of spatial vision in the 5 dark-reared animals from which measurements were made.

#### Development of Grating Visual Acuity

In earlier studies (Timney et al., 1978; Mitchell, 1988) of animals dark-reared for long periods (to 12 months of age) spatial vision was shown to emerge very slowly at a rate that decreased with the length of the deprivation and only in animals deprived to 4 months of age or less did the visual acuity achieve the level of typically reared adults. The results of longitudinal measurements of the acuity of 5 dark-reared animals upon emergence from the darkroom are displayed in Figure 3. Different colors are used to depict the data from animals from the two litters with blue used to represent data from C115 and C116 while data from the second litter are shown by orange symbols (C127-129). For 1–2 days after emerging from the dark, animals appeared blind (designated by the letter “B” in Figure 3) on the jumping stand because they appeared to employ only tactile cues such as reaching from one side or the other for the closed door with their paws to locate a closed from an adjacent open door on the jumping stand (Mitchell, 2013). The first indication that the kittens were able to locate the closed door by visual cues alone occurred after 1 (C128), 3 (C115, C116, and C129) or 5 (C127) days and was designated as an “open door” discrimination (OD) in Figure 3. The mean time (3 days) for this initial sign of vision to emerge on the jumping stand is consistent with data and predictions (5 days) based on an earlier study (Mitchell, 1988; Figure 7). Because this discrimination could in theory be solved by luminance cues alone, the operational indication of the acquisition of spatial vision was assumed to coincide with the ability to discriminate a vertical from a horizontal grating, a task passed on average 2.5 days later, or 5.5 days after animals were first exposed to light. Thereafter the acuity improved very gradually to stabilize at normal age-matched levels after 3 months in the light at about 4 1/2 months of age (Figure 3). Comparison of the time course of development of visual acuity in normal kittens (Giffin and Mitchell, 1978; Figure 2) permits an additional perspective to be placed on the slow improvement of the acuity of the dark-reared kittens. After a month in the light, when they were 10 weeks old, the latter kittens had achieved an average acuity of only 1.5 cycles/deg, or nearly one-quarter that of normal kittens of the same age.

#### Overall Motility

The mobility of kittens was assessed in terms of the number of line crossings made during a 5-min videotaped period in the arena. Figure 4 shows the results of these measures for the 3 light-reared kittens and representative data from one (C116) of the dark-reared animals plotted as cumulative frequency distributions as is common in studies with rodents. All kittens began moving around the apparatus slowly and cautiously as a possible consequence of the novelty of the new environment. For more than a week the normal kittens crossed less than 5 lines in 5 min but at about 3 weeks of age. the number of lines crossed each day tripled for two animals who were littermates (C117, C118). By contrast, the activity of the third normal kitten (C119) from another litter was consistently lower and delayed by approximately a week in comparison to the data from the other two. In addition to its origin from another litter, C119 was exposed to light earlier (at birth) in comparison to the other two kittens. The rapid increase in locomotion exhibited by C117 and C118 suggest that this occurred despite their having received only 1–3 h of light exposure each day. Their mobility became more deliberate as they grew accustomed to the arena, at which point play behavior began. Kittens appeared to react to imaginary objects and struck at the lines drawn on the arena floor. By comparison, the dark-reared animals were immediately very active upon their initial exposure to light, although it is important to note that this exposure occurred at an older age (6 weeks). Daily line crossing data was collected from only one dark-reared animal but data from the first 3 days in the light for two others (C114, C115) was almost identical. The high overall motility of the dark-reared animals appeared to reflect their age rather than with the limited amount of light exposure they had received. Interestingly, the normal light-reared animal, C119, that received the greatest daily exposure to light, was less mobile than the other two light-exposed kittens, suggesting that only a short period of light exposure (1–3 h) per day in the period from P10 to P42 may be sufficient for kittens to develop a high degree of mobility.

#### Visuomotor Responses to Pendulums

##### Following Behavior

Visuomotor development was tested in terms of the response to pendulums of two different sizes held on nylon or regular strings and swung in front of the kitten’s face. The behavior exhibited by kittens during the first 15 s of recorded attention to these stimuli was scored for the time they spent following the stimuli with their head and eyes (gaze), and the amount of time they attempted to strike at them. Only the first 15 s of this behavior was scored since the kittens appeared to engage with the stimuli in bursts of about this length. Cumulative following times (Figure 5, top) for the large pendulum as a function of age demonstrated that light-reared kittens began following pendulums at between 25 and 30 days of age, while dark-reared kittens followed either pendulum within a day of their initial exposure to light at 42 days of age. While the overall following behavior of the 3 light-reared animals was similar, the kitten that received the longest daily visual exposure (C119) began following the large pendulum earlier (by 4 days) than did the others. Interestingly, the initial responses of the 3 light-reared kittens to the small pendulum (Figure 4, bottom) began at the same age (at ∼ 30 days of age). Thereafter the daily amount of gaze following increased rapidly in the next 10 days followed by a slower increase to reach an asymptote at about 70 days of age. Despite differences between the amount of daily light exposure received by the 3 light-reared kittens, the acquisition of gaze following behavior followed a closely similar path for all animals, a result that suggests that only short periods of light each day (1–3 h) was sufficient for normal development of following behavior.

Undoubtedly. the most remarkable result was the fast emergence of gaze following responses to the pendulums by the 3 dark-reared kittens that began in the first 2 days of exposure to light at a time when they exhibited no sign of spatial vision on the jumping stand (Figure 3) an ability that was not achieved for on average 5.5 days following their introduction to light. Furthermore, the subsequent pattern of following behavior was remarkably similar to the progression observed in light reared kittens following emergence of this behavior albeit at a younger age.

A more detailed description of the following behavior of the dark-reared kittens is provided by analysis of the accuracy of the gaze responses obtained through frame by frame analysis of the video records. Representative results from analysis of the video records of the gaze responses to motion of the large pendulum for one dark-reared kitten (C114) made on its second day in the light are shown in Figure 6. The blue symbols show plots of the gaze responses to motion of the pendulum which is indicated by the gray symbols. Even though following responses to the pendulum at that stage were quite infrequent, the few gaze responses that did occur were quite accurate in terms of their amplitude and speed, albeit with a slight temporal delay. Results from a littermate (C115) made after an extra day in the light are displayed in Figure 7 and exhibit a somewhat longer delay and reduced amplitude of the gaze movements with respect to the motion of the pendulum. These following responses occurred at a time when C115 was designated as blind on the jumping stand. The following responses observed in the first few days in the light were somewhat variable as a possible reflection of fluctuation in the degree of engagement with the stimuli after several repetitions of the regular pendulum arc of motion.

##### Striking Behavior

**FIGURE 8:**
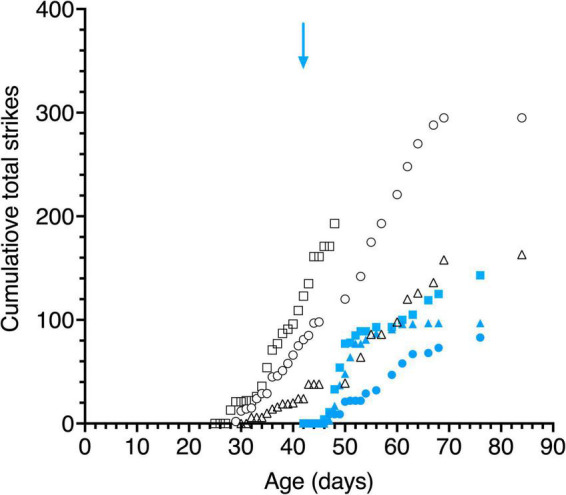
Cumulative total strikes to the swinging small pendulum in daily 15 s episodes as a function of age. Data are displayed for 3 light- exposed (open black symbols: C117 triangles, C118 circles, C119 squares) and 3 dark-reared kittens (blue filled symbols: C114 triangles, C115 circles, C116 squares). The blue arrow indicates the age of the dark-reared kittens when they were first exposed to light.

Large variability was observed between the kittens in their striking behavior, especially for the large pendulum and one kitten (C115) responded only to the small pendulum. All kittens struck at the smaller pendulum first, possibly because they may have been intimidated by the larger one. Because of the much larger variability of striking observed among animals with the large pendulum, striking behavior of all kittens is displayed in Figure 8 for only the small pendulum. Although the dark-reared kittens followed the small pendulum after 1–3 days in the light, striking responses occurred on average 2 days later. Light reared kittens struck at the small pendulum on the same or the next day (C117) as their following responses were first observed.

##### Responses to Moving Laser Spots

**FIGURE 9:**
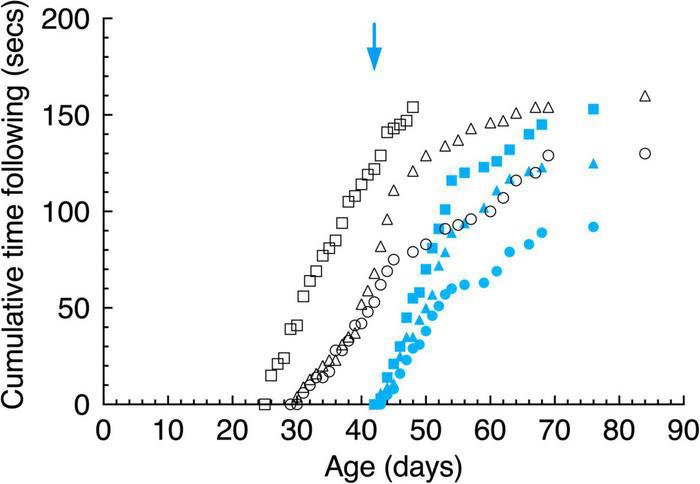
Cumulative gaze following behavior of a moving laser spot in daily 15 s episodes as a function of age. Data are displayed for 3 light-exposed (open black symbols: C117 triangles, C118 circles, C119 squares) and 3 dark-reared kittens (blue filled symbols: C114 triangles, C115 circles, C116 squares). The blue arrow indicates the age of the dark-reared kittens when they were first introduced to light (at P42 days). The blue arrows indicate the age of the dark-reared kittens when they were first exposed to light.

Robust following gaze responses were observed to moving laser spots (Figure 9) with a similar time course of development as that observed with moving pendulums (Figure 5). However, a comparison of the ordinates of Figure 9 with Figure 5 reveals that all animals consistently followed pendulums for longer than laser spots, a possible reflection of a preference for following solid objects by kittens in the age-range tested. As with pendulums, the dark-reared animals followed moving laser spots almost immediately after exposure to light. Another consistent observation was that kittens preferred the movement of hand-held lasers as compared to spots moved under computer control. A likely explanation for this preference was the regularity of the linear or sinusoidal motion of the latter as opposed to the unpredictable movements of the hand-held laser.

With the exception of C118 for whom initial strikes to both stimuli occurred at the same age, all kittens stuck at moving laser spots 1 to 3 days earlier than they did at moving pendulums. This preference may arise from the small size of the laser spot (5 mm dia.) which may have made it less intimidating than even the moving solid small pendulum (28 mm dia.). Although detailed analysis of the trajectory and accuracy of the striking responses of the dark reared kittens to either laser spots or pendulums were not made because of the slow video frame rate employed, it was apparent that this merits further study as the nature of these responses changed with exposure to light. At first the striking responses were slow and tentative and appeared to be stereotypical as they appeared directed toward a target at a constant distance from the animal irrespective of its actual distance. These observations confirmed those made earlier by Vital-Durand et al. (1976).

### Monocularly Deprived Animals

Preliminary observations were made on two kittens of their visuomotor responses to moving pendulums or laser spots in the days surrounding termination of a 3-week period of monocular deprivation that began at P30. Data on visual following and striking at the stimuli were collected in the first minute after the kitten was placed in the arena. Before termination of the period of MD, the visuomotor responses of both kittens reflected the ability of the non-deprived eye. However, after the period of MD, one animal (C480) was reverse occluded so that it was forced to exclusively employ its formerly deprived eye. By contrast, C479 had both eyes open after the period of MD so that tests of the visual abilities of the formerly deprived eye were made with a hard opaque contact lens placed on the cornea of the fellow eye (Mitchell, 1988). Whereas the kittens actively followed and struck at the pendulum for about 50 s of the 1 min of exposure to the moving pendulum using the non-deprived eye on the day before the end of the period of MD, in the next 6 days using the formerly deprived eye they only followed the pendulum if it was dragged across the floor of the arena to provide an auditory cue to its changing location. Once lifted above the floor, the kittens appeared unable to follow the pendulum at all. On the seventh day one kitten (C479) followed the pendulum for 5 s and struck at it 4 times when using the formerly deprived eye. Because these two kittens participated in another experiment it was not possible to follow any possible further recovery. This very preliminary result suggests that a 3-week period of monocular deprivation had a far greater impact on gaze following and striking than did a much longer period of dark-rearing.

## Discussion

This study was designed as a preliminary exploration of the use of a simple arena to allow longitudinal recording of changes in visuomotor behavior in normal kittens as well as in kittens that had experienced two forms of selected early visual deprivation. In addition to documenting increases in general locomotion in normal kittens from 10–12 days of age, changes in their reaction to visual stimuli in terms of gaze following and striking responses were studied from about 25 days of age. Normal kittens showed a regular increase in locomotion in the arena beginning at about 3 weeks of age, and approached an asymptotic level at about 6 weeks. Within the small sample there was evidence of considerable variability in both the onset and the rate of increase of motility. Dark reared kittens showed a high degree of motility on immediate introduction to light which the limited data suggested increased subsequently at a rate similar to that of normal kittens once they became mobile in the arena at about 3 weeks of age.

Several salient points emerged from studies of the responses of normal kittens to moving solid objects (pendulums) or laser spots. First, gaze following movements to the pendulums and to laser spots began at between 25 and 30 days of age followed by a rapid and steady increase to an asymptotic level at about 70 days. On any day, normal kittens followed pendulums for longer than for laser spots, indicating a possible preference for solid objects. Second, striking responses to pendulums with their paws began on or within a day of initiation of gaze responses. Kittens struck at the small pendulum more than they did to the large pendulum and one kittens did not strike at the latter at all. Third, striking responses to moving laser spots began either on the same day or as much as 3 days earlier than strikes to pendulums.

Study of the two groups of selectively visually deprived kittens in the arena revealed some important differences between the immediate consequences of the two forms of deprivation on visuomotor behavior. Whereas monocular deprivation had a profound effect on motility as well as on the following and striking responses to moving stimuli when kittens were forced to use their deprived eye, the consequences of a period of darkness (binocular visual deprivation) were far less. A very salient finding of this study was the almost immediate acquisition of gaze following movements of the head and eyes to moving stimuli of the dark-reared kittens on first exposure to light that were accompanied by an equally fast emergence of striking responses. The fast emergence of visuomotor responses stood in clear contrast to the very slow recovery of spatial acuity in the same animals over many months This divergence was especially apparent in the first few days after emergence from the darkroom at which time on the jumping stand kittens could only find a closed door by use of tactile cues and appeared incapable of jumping at all to a visual stimulus. For several more days, even after they were able to step or jump from a few centimeters above the stimuli, they were unable to discriminate a vertical from a horizontal grating suggesting that they may possess only the ability to make luminance discriminations. By contrast, gaze following behavior in the dark reared kittens was evident immediately upon exposure to light with a very similar time course to the maturation of such behavior in light-reared kittens once it began at 25–30 days of age, suggesting that the maturation of this behavior in dark-reared kittens was triggered immediately upon their introduction to light. A similar conclusion was reached by Van Hof-Van Duin (1976) on the basis of an array of different tests.

The rapid emergence of responses to moving stimuli in the dark-reared kittens suggests that neurons and pathways involved in the analysis of motion for visuomotor responses may mature fast and be partially or completely immune to disruption by early binocular visual deprivation. Evidence accumulated over decades (Schneider, 1969; Goodale and Milner, 1992) suggests that the analysis of motion and the execution of motor action toward stimuli in space are processed in a separate processing stream to that involved in the analysis of spatial form. In humans, non-human primates and cats (Lomber et al., 1996), these are identified, respectively, as the parietal and temporal cortical processing streams. In addition, initial orienting toward the visual stimuli and the gaze responses to their movement suggests involvement of the superior colliculus. Neural structures of the temporal visual pathway that are known to be involved in the analysis of fine spatial detail, are extremely vulnerable to disruption by early visual deprivation (Mitchell and Maurer, 2022) which would explain the more severe and protracted deficits in spatial acuity. Although less is known of the effects of visual deprivation on either the superior colliculus or the parietal processing stream in the cat, there is some evidence to suggest that the effects of deprivation may differ for the two cortical visual processing streams. For example, the profile of the sensitive period of vulnerability to the effects of early monocular deprivation in the cat lateral suprasylvian cortex is shorter than that for the visual cortex (Jones et al., 1984). In contrast to cells in the primary visual cortex, cells of the lateral suprasylvian cortex appear to be functionally specialized for motion processing and in general the development of the stimulus response properties of these cells begins earlier and proceeds faster than the stimulus selectivity of cells in the primary visual cortex (Price et al., 1988).

With respect to the profound effects of monocular deprivation on the visuomotor responses of C479 and C480, it has been known for some time that this form of deprivation exerts substantial effects on the response properties of cells in the superficial layers of the superior colliculus (Hoffmann and Sherman, 1974) and in addition, promotes substantial shifts of ocular dominance in the lateral suprasylvian cortex (Jones et al., 1984) to favor the non-deprived eye. Thus, the known effects of monocular deprivation on the superior colliculus and on the parietal visual pathway provide a ready explanation for the substantial loss of visuomotor responses when the monocularly deprived kittens viewed stimuli with their deprived eye. By contrast, the smaller deficits observed with dark-reared kittens suggest that cells in either the superior colliculus or in the lateral suprasylvian cortical areas may not have been effected by this form of early visual deprivation.

Future studies with the methodology employed here must include the ability to manipulate the speed of the visual targets whether they be solid objects or moving laser spots as anecdotal observations suggested that kittens made gaze movements more frequently to stimuli moved slowly and with a certain degree of unpredictability to their path. Such tweaks to the methodology would facilitate study of the temporal characteristics of the following and striking responses to solid stimuli and to laser spots at different times following early visual deprivation. In particular it will be of interest to determine how the strike trajectory and accuracy of these responses evolves with time during recovery. Relevant to this work were observations made many years ago on kittens reared without sight of their limbs (Hein and Held, 1967) of a gradual evolution of visual placing responses with time into elicited versus guided components.

## Data Availability Statement

The raw data supporting the conclusions of this article will be made available by the authors, without undue reservation.

## Ethics Statement

The animal study was reviewed and approved by the University Committee on Laboratory Animals, Dalhousie University.

## Author Contributions

KM, DM, and KD collected the data. KM and AM analyzed the data. DM and KD contributed in concepts and writing of the manuscript. All authors contributed to the article and approved the submitted version.

## Conflict of Interest

The authors declare that the research was conducted in the absence of any commercial or financial relationships that could be construed as a potential conflict of interest.

## Publisher’s Note

All claims expressed in this article are solely those of the authors and do not necessarily represent those of their affiliated organizations, or those of the publisher, the editors and the reviewers. Any product that may be evaluated in this article, or claim that may be made by its manufacturer, is not guaranteed or endorsed by the publisher.
